# History of Bioelectrical Study and the Electrophysiology of the Primo Vascular System

**DOI:** 10.1155/2013/486823

**Published:** 2013-07-09

**Authors:** Sang Hyun Park, Eung Hwi Kim, Ho Jong Chang, Seung Zhoo Yoon, Ji Woong Yoon, Seong-Jin Cho, Yeon-Hee Ryu

**Affiliations:** ^1^Biomedical Team, KAIST Institute for Information Technology Convergence, Korea Advanced Institute of Science and Technology (KAIST), Daejeon 305-701, Republic of Korea; ^2^Department of Anesthesiology and Pain Medicine, College of Medicine, Korea University, Seoul 136-705, Republic of Korea; ^3^Impedance Imaging Research Center & Department of Public Administration, Kyung Hee University, Seoul 130-701, Republic of Korea; ^4^Acupuncture, Moxibustion & Meridian Research Center, Division of Standard Research, Korea Institute of Oriental Medicine, Daejeon 305-811, Republic of Korea; ^5^Medical Research Division, Acupuncture, Moxibustion and Meridian Research Group, Korea Institute of Oriental Medicine, 1672 Yuseongdae-ro, Yuseong-gu, Daejeon 305-811, Republic of Korea

## Abstract

*Background*. Primo vascular system is a new anatomical structure whose research results have reported the possibility of a new circulatory system similar to the blood vascular system and cells. Electrophysiology, which measures and analyzes bioelectrical signals tissues and cells, is an important research area for investigating the function of tissues and cells. The bioelectrical study of the primo vascular system has been reported by using modern techniques since the early 1960s by Bonghan Kim. This paper reviews the research result of the electrophysiological study of the primo vascular system for the discussion of the circulatory function. We hope it would help to study the electrophysiology of the primo vascular system for researchers. This paper will use the following exchangeable expressions: Kyungrak system = Bonghan system = Bonghan circulatory system = primo vascular system = primo system; Bonghan corpuscle = primo node; Bonghan duct = primo vessel. We think that objective descriptions of reviewed papers are more important than unified expressions when citing the papers. That said, this paper will unify the expressions of the primo vascular system.

## 1. Primo Vascular System

The primo vascular system has been studied as part of the anatomical and histological research of the last 10 years. It is a novel thread-like structure that remains unrevealed in animals and humans. Particularly, the primo vascular system was selected as the feature article and for the cover page in the Journal of Anatomical Record [1]; it provoked controversy in the anatomical society and among acupuncture scientists. Since then, the discovery of the primo vascular system in lymphatic vessels [2], cardiac vascular vessels [3], and the brain [4] has received much attention.

Early scientists of the primo vascular system, who focused on anatomical and histological studies, hypothesized about the relationship between oriental medicine and acupuncture and the meridian. These findings are helping to integrate the theories of conventional and alternative medicine [5].

Kim, who initially studied the anatomical structures of the acupuncture and the meridian, declared that “This project developed from our inherited oriental medicine, which is our ancestors' creative and noble endeavor as approved at the third Joseon Labor Party Congress” in his first paper [6].

Moreover, Soh considered that Bonghan corpuscles and ducts were identical to acupuncture sites and meridians [5], stating his theory that the Bonghan circulatory system may be an extension of acupuncture and meridians [7]. It was reported that DNA was in the primo vascular system [1]; evidence showing that the primo vascular system may be a DNA circulatory system has been reported as well [4].

Electrophysiology is a science of the electrical properties of biological tissues and cells. It involves measurements of voltage changes or electric current on a wide variety of scales from single-ion channel proteins to whole organs such as the heart. In this paper, we introduce the history of electrical signals and the electrophysiology of the primo vascular system.

Section 2 introduces the understanding of electrophysiology and modern techniques to measure the bioelectrical signal for general readers.  Section 3 describes the beginning of the bioelectrical study of acupuncture meridian briefly and reviews the electrophysiological study of the primo vascular system past and present. The last section arranges the result of the reviewed papers and suggests the future work for the electrophysiology of the primo vascular system.

## 2. Development of Electrophysiology with Electrical Signal and Basic Medicine

### 2.1. Bioelectrical Signal Studies and Electrophysiology

**Figure 1:**
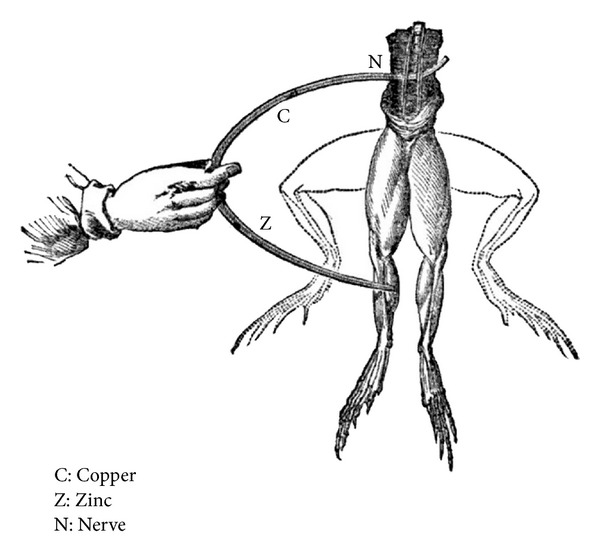
The electrical stimulation of a frog nerve. It was found that the current flows through the two different metals, coming into contact with animal muscle.

In 1791, the Italian physician and physicist Luigi Galvani first recorded the phenomenon of electrical signals while dissecting a frog on a table where he had been conducting experiments with static electricity (Figure 1). Galvani coined the term animal electricity to describe the phenomenon, while contemporaries labeled it galvanism. Galvani and his contemporaries regarded muscle activation as resulting from an electrical fluid or substance in the nerves [8, 9].

**Figure 2:**
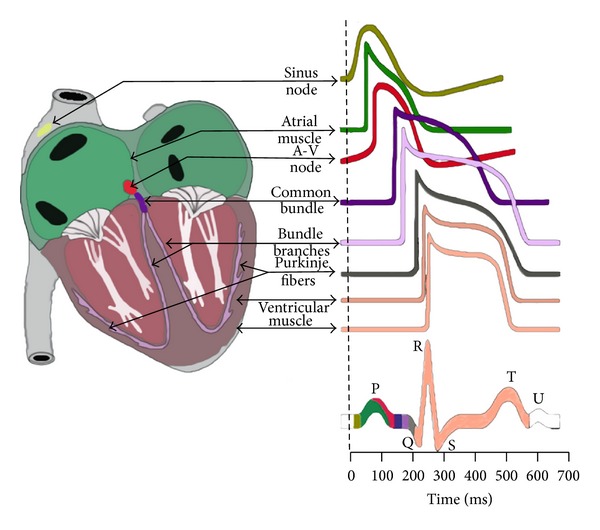
Electrophysiology of the heart.

In the work to study cardiac electrophysiology in order to gain a better understanding of bioelectricity, cardiac electrophysiology emerged as an important area to elucidate and diagnose the heart function and to treat heart disease, including arrhythmia, through electrocardiograms (Figure 2) [10]. The function of tissues and organs in humans is closely related to analyses of the bioelectrical signals from these features.

### 2.2. Development of a Measurement Method for Bioelectric Signals

**Figure 3:**
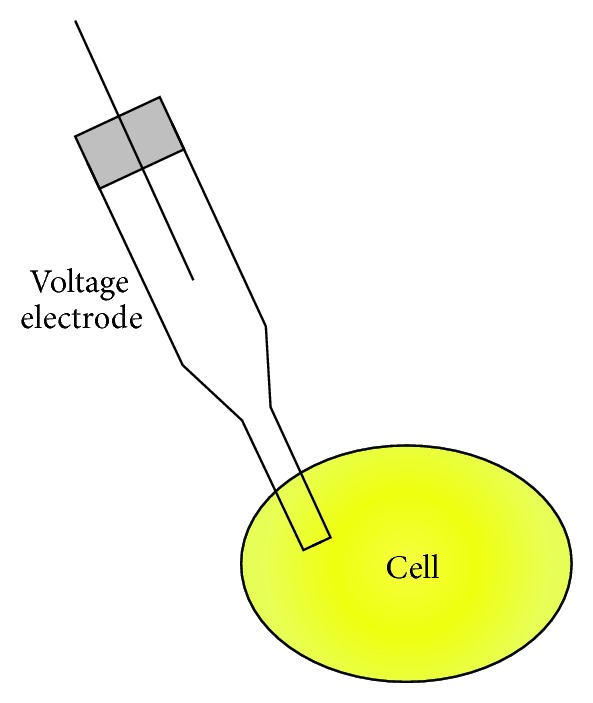
Intracellular recording technique.

Electrophysiological studies, which started from frog muscle response research, extended the fundamental knowledge of nerve cells through squid axon membrane potential measurements using an electrode when Hodgkin and Huxley began this work in 1952 [11]. Graham and Gerard succeeded to create and develop an electrode with a finer diameter of 2–5 um to measure the intracellular potential (Figure 3) [12]. With the development of this measurement technique, Neher and Sakmann reduced the signal-to-noise ratio using a seamless seal between the cell membrane and the electrode, establishing the foundation of the study of ion channels in the cell membrane [13]. Cell membrane potential measurement techniques in electrophysiological research are outlined later. 

#### 2.2.1. Intracellular Recording (Voltage Clamp and Current Clamp)

An electrical signal representing cell activities is a result of the activity of the ion channels in the cell membrane. The cell activity is associated with whether the cell membrane ion channel is activating or not and is then related to the electrical signals from the cells. Related to this, the voltage clamp technique was developed to measure the intracellular ion flow with the constant membrane potential. This technique was useful in research on the mechanism of biological electrical signals generated by the ions such as K^+^ or Na^+^ passing through the electrically opened and closed channels [11–13].

**Figure 4:**
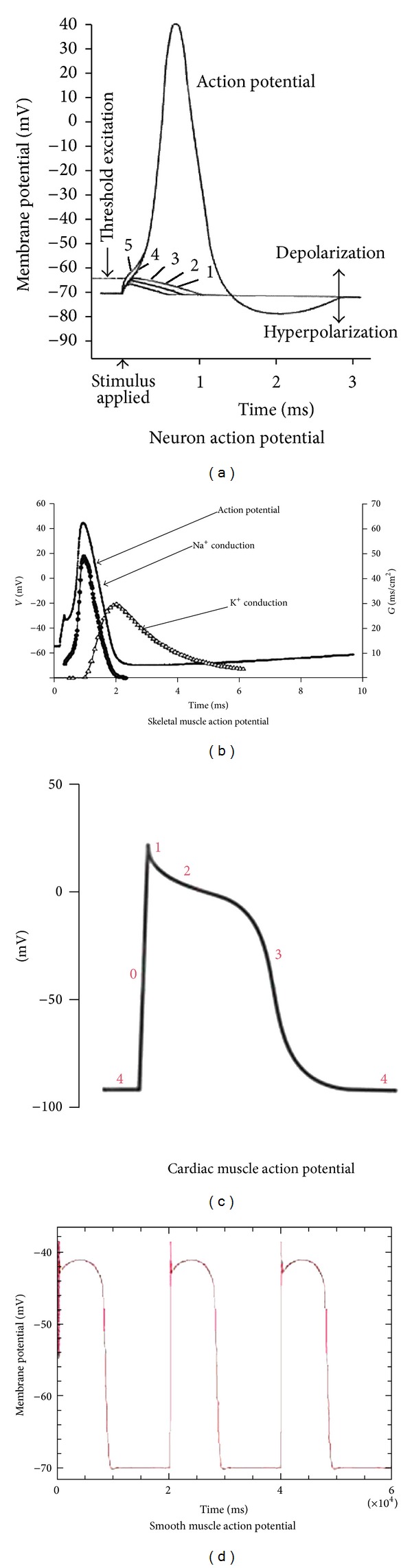
A comparison of the action potentials of major tissue cells.

In contrast, the current clamp was used to measure the changes of the voltage inside the cell by means of a constant current; this was useful to classify cell types by understanding the action potential of the cells (Figure 4) [14, 15]. 

#### 2.2.2. Patch Clamp

**Figure 5:**
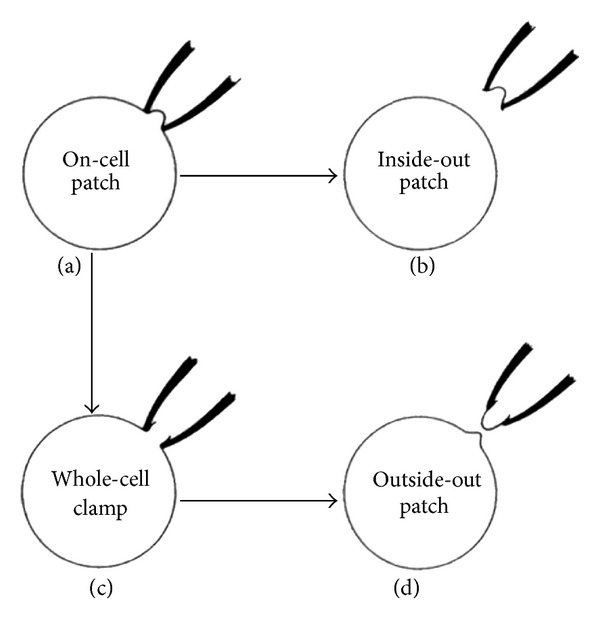
Patch clamp technique.

As an intracellular recording technique which involves inserting an electrode into a cell directly, the patch clamp technique was created for the purpose of separating and analyzing of each ion channel existing on the membrane of a cell. Patch clamp recordings use, as an electrode, a glass micropipette that has an open tip diameter of about one micrometer (1 *μ*m), which is a size enclosing a membrane surface area or “patch” that often contains just one or few ion channel molecules. This type of electrode is distinct from the “sharp microelectrode” used to impale cells in traditional intracellular recordings, in that it is sealed onto the surface of the cell membrane rather than inserted through it. Many researchers, including Neher and Sakmann, studied ion channels in various cells using the patch clamp technique (Figure 5) [16–18].

They observed that the electrophysiological characteristics in each major tissue cell, especially the resting potential, showed a different form (Table 1). This cell-size level technique led to the development of electrophysiology and has produced many eminent scientists and Nobel Award winners [19–22].

The development of the measurement technique such as intracellular recording and patch clamp advanced the electrophysiological research.

## 3. Beginning of Electrophysiology of the Primo Vascular System

Although the primo vascular system has been studied intensively over last 10 years, there is no complete evidence of whether the primo vascular system is an extension of acupuncture and meridians system or is identical to acupuncture and meridians, which are key concepts in oriental medicine. Oriental medicine scientists scarcely accept hypotheses pertaining to the anatomical structures of acupuncture and meridians. However, in their work on the primo vascular system, Soh [7] and Lee et al. [23] declared that they were inspired by Bonghan Kim's theory from the 1960s [6, 24–27]. Therefore, the beginning of the electrophysiology of the primo vascular system established a standard from the beginning of the measurement of the electrical properties related to acupuncture and meridians.

### 3.1. Historical Trends of the Bioelectrical Study of Acupuncture and Meridians

The bioelectrical signal measurements of acupuncture and meridians were attempts to determine the existence of these structures. These studies were done using animals and cadavers; it was assumed that human study was only partially complete. 

Jeh found that acupuncture points had lower resistance than the surrounding skin by measuring the skin resistance [28]. Overhof verified that the resistance at acupuncture points was lower than that of nonacupuncture points [29]. Later, Ogata et al. reported that acupuncture points had lower resistance than non-acupuncture points located in the same meridian [30]. In particular, Niboyet's methods have led to the development of ear acupuncture, which has become one of the acupuncture treatment therapies offered in Europe. Considering this low-resistance feature, Voll designed an acupuncture diagnostic apparatus using microcurrents [31].

Studies of the low-resistance properties of acupuncture points have been performed intermittently. Recently, Ahn et al. took ultrasonic images of acupuncture points and proposed, anatomically, that a collagenous band in connective tissues was related to the low-impedance property of acupuncture points [32].

A modern system to be able to measure the resistance of acupuncture point has been developed recently based on the result of the lower resistance on the acupoint [33].

But there are still limitations to measure the special point on the skin by classical theory and method since 1950s.

### 3.2. Bioelectrical Study and Electrophysiology of the Primo Vascular System in the 1960s

Kim published five papers in total about the existence of the anatomical structure of meridians at the Kyungrak Institute in the 1960s [6, 24–27, 34]. Three out of the five papers described electrical signal measurements and analyses of acupuncture points and meridians. His first paper investigated the electrical properties of acupuncture points and meridians. The following description was extracted from his first paper, given at the conference of the Pyongyang Medical School on October 18, 1961:

*“This project was started to develop and propagate oriental medicine, which is our ancestor's creative and noble endeavor as approved at the third Joseon Labor Party Congress… We have set ourselves an assignment to find the electrical properties of meridians first and then the Kyungrak system based on these properties” [6].*

Kim already knew about research reports on the electrical resistance of acupuncture points, showing that it was lower than the areas around the spots. He had attempted to measure and examine unseen singular points on the skin. When writing his paper, he investigated the electrical properties of acupuncture by measuring the resistance and voltage around the acupuncture points of rabbits. The resistance value was about 20,000–80,000 Ω when applying 100 *μ*A, and it was lower than the values around the point. He indicated the problem of the changing measurement value in terms of the measurement time, interval, and number of measurements. However, the locations of the low-resistance points were fixed; the distribution of the locations coincided with the acupuncture points described in the Dongui Bogam, which is a physician Book of Traditional Medicine compiled by Heo Jun in 1613 during the Joseon Dynasty of Korea [35].

**Figure 6:**
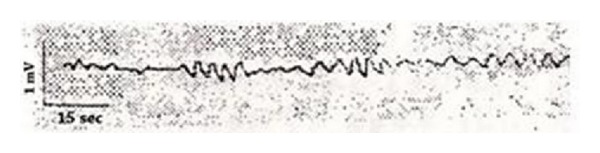
The electric induction on a Nogung acupuncture point (PC8).

**Figure 7:**
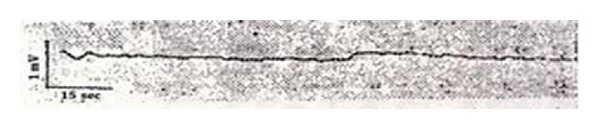
The induced electricity at non-acupuncture points located 1 cm from Nogung.

It was surprising that he had found that the voltage value of the acupuncture point changed consistently. The voltage change was a regular and rhythmical wave group with a wave period of 3–6 sec and an intensity level of 0.1 mV. He reported that 5–7 waves were detected continuously, followed by a resting phase (Figure 6). He asserted that non-acupuncture points did not display this phenomenon (Figure 7).

He considered that the changing values of acupuncture points may be connected to physiological properties and therefore ran an interrelationship experiment.

**Figure 8:**
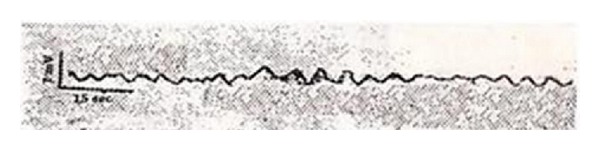
The induced electricity at an acupuncture point after hyperkinesis of the large intestine.

It was an experiment to measure the interactional electrical signals between stimulation by a needle and large intestine movements. He reported some interaction between acupoint ST_36_ and the movement of the large intestine when measuring the electrical signals and stimulating the intestine (Figure 8).

**Figure 9:**
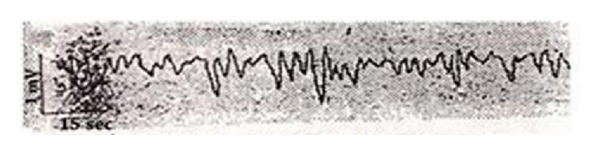
The electric induction on the Susamni acupuncture point (LI10).

His interpretation of the result from his interaction experiment was different from those of general acupuncture scientists at that time. He suggested that acupuncture and internal organs should be connected materially to each other, hypothesized that acupuncture points and meridians would be anatomical structures in the human body, and started to explore anatomical tissues (Figure 9).

B. H. Kim advanced anatomical and histological research and Kyungrak circulatory research by developing his first paper into his second published paper, entitled On the Acupuncture Meridian System.

He named the discovered anatomical structures around acupuncture points Bonghan corpuscles and the thread-like structure connected to Bonghan corpuscles Bonghan ducts. (He did not use the word Bonghan system. He only named the new anatomical and histological structures of Kyungrak system Bonghan corpuscles and Bonghan ducts [24].)

In order to investigate the circulation of the Kyungrak system, he analyzed the physiological and bioelectrical measurements of Bonghan corpuscles first, as this was the basis of the contention that the morphological characteristics of Bonghan corpuscles were smooth muscles and secreting cells.

He inserted an electrode directly into a Bonghan corpuscle and measured the bioelectrical signal from Bonghan corpuscle. It was very similar to the extracelluar recording method in the modern technical term. But there were limitations to be able to measure the bioelectrical signal from the tissue of Bonghan corpuscle because of noises. Now modern technique to measure the bioelectrical signal can measure the electrical signal from single cells [36].

The physiological study of Bonghan corpuscles and ducts progressed as the manner in which bioelectrical signals changed by various stimulations was studied.

He proposed that Bonghan corpuscles and ducts responded to various stimuli and that the responses were similar to those of nerves. There were many experiments conducted to determine where the signal was conducted—whether it was along the Bonghan corpuscle and duct or another area [6].

**Figure 10:**
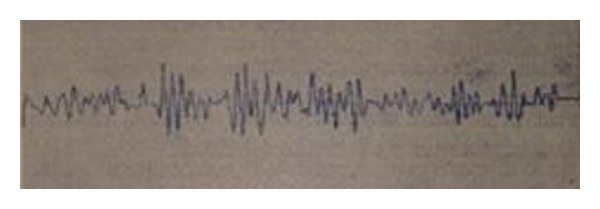
The bioelectrical activity from a Bonghan corpuscle.

The bioelectrical research into Bonghan corpuscles recorded the changing values of the bioelectrical signals by inserting an electrode into a Bonghan corpuscle. The experimental result reported that the change of the potential in a Bonghan corpuscle was similar to a sine curve, terming this curve a “*ㄱ*” (geu) wave with a period of 3–6 seconds. Also identified was a “*ㄴ*” (neu) wave at 7–10 seconds and a “*ㄷ*” (deu) wave at 20–25 seconds. The amplitude of each wave was 0.1 mV (Figure 10).

Particularly, these electric potential changes disappeared when the temperature of the environment dropped below 27°C but reappeared when the temperature was raised to 39°C immediately. He suggested that the bioelectrical properties of acupuncture points as explained by the nervous system were incorrect, as the phenomena of the changing signal appeared from Bonghan corpuscles, which are not related to the nervous system.

The next experiment involved assessing the excitability and response of Bonghan corpuscle by various stimuli. B. H. Kim expressed that “*the study of excitability of Bonghan corpuscles was a basic problem to elucidate the physiological functions of the Kyungrak system.*” Acetylcholine and pilocarpine were used and the electrical potential changes were recorded.

**Figure 11:**
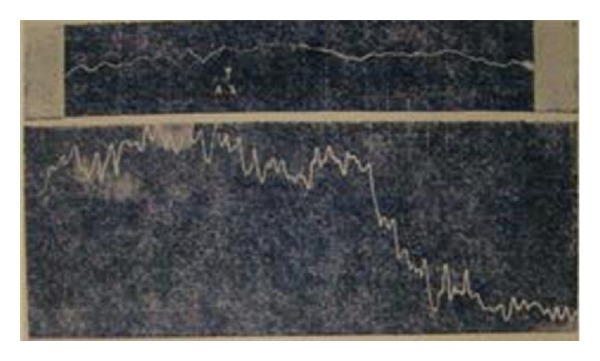
The changes in the bioelectrical activity of a Bonghan corpuscle after an injection of acetylcholine.

**Figure 12:**
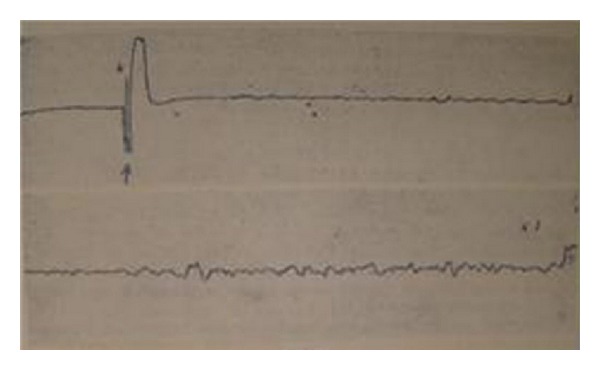
Strong electrical stimulation amplifies the changes in the bioelectrical activity of a Bonghan corpuscle.

The experiment result reported that the bioelectrical signals changed after stimulation with acetylcholine, pilocarpine, and Novocain. He noted that the signals varied according to the different types and concentration of drugs (Figure 11). Finally, the experiment of the bioelectrical signal conduction of the Kyungrak system was based on his first paper, which reported the interaction materially and functionally between Bonghan corpuscles and internal organs (Figure 12) [6]. This study was significant in that it determined how the stimulation signals of Bonghan corpuscles were delivered in the Kyungrak system. He stimulated Bonghan corpuscles connected to a Bonghan duct to measure the bioelectrical potential of the next Bonghan corpuscle. The experimental result reported that the bioelectrical potential changed after a certain time and that the delivery speed of the effect stimuli was 3.0 mm/sec at a superficial Bonghan corpuscle (a Bonghan corpuscle in the skin).

The relationship between mechanical movements and the bioelectrical signals of Bonghan ducts was reported in his third paper. It started with an exploration of Bonghan ducts, not Bonghan corpuscles, and developed this line of research from previous results. The properties of bioelectrical signals were similar to those of a superficial Bonghan corpuscle (a Bonghan corpuscle in the skin), and the delivery speed was 1–3 mm/sec for communication in both directions. He asserted that the phenomena of longitudinal periodic lateral and pulsatory mixed movement could be observed [25]. The movement delivery speed of the Bonghan duct was 0.1–0.6 mm/sec, which was much slower than that of the bioelectrical signals. However, the experimental results pertaining to the relationship between the mechanical movement and bioelectrical changing were limited because the explanation of the experiment setup and data was insufficient. Nonetheless, he insisted that there was convincing evidence of the flow of liquid actively through Bonghan ducts. Afterward, his study concentrated on Sanal cells (primo microcells) and the liquid through Bonghan ducts. 

## 4. Recent Bioelectrical and Electrophysiology Research on the Primo Vascular System

**Figure 13:**
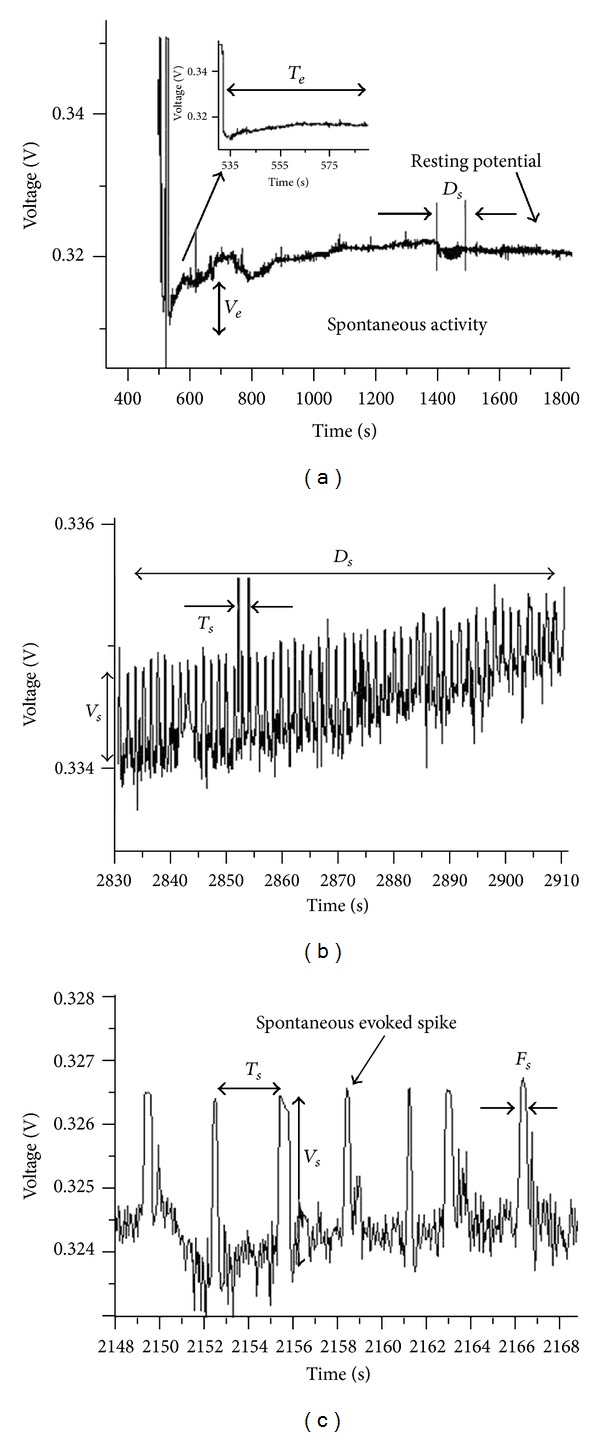
Resting potential and spontaneous activity of the potential in a Bonghan corpuscle (BHC): (a) at the moment of the microcapillary insertion into a cell membrane of a BHC. The potential decreases abruptly by about 38 mV from the reference potential of the bath. *V*_*d*_ is the potential drop. The potential increased slowly to the resting potential by 6.7 mV. *V*_*e*_ is the small increase, and *T*_*e*_ is the time of the increase (45.2 sec). The resting potential remained stable with fine background fluctuations, and the irregular activity of spontaneously evoked spikes in the resting potential arose for a duration (*D*_*s*_) of about 16.6 seconds. (b) The spontaneous activity in the resting potential was examined more closely. The average amplitude (*V*_*s*_) is 1.1 mV, and the average period (*T*_*s*_) is 0.8 sec. (c) More magnified view of the spontaneous activity. A spike has an average half-width (*F*_*s*_) of 0.27 seconds.

Electrophysiological research on the primo vascular system halted after his third paper. His fourth and fifth papers were focused on flowing Bonghan liquids and Sanal cells through Bonghan corpuscles and ducts. Park started to measure and analyze the electrical potential of Bonghan corpuscles in the 2000s (Figure 13) [37]. Various experiments involving the staining Bonghan corpuscles and ducts were attempted to distinguish them from other tissues [4, 23, 38–42]. Lee et al. reported that trypan blue was the most effective type of stain for these corpuscles and ducts in 2007 [4]. The trypan blue staining method was advanced by B. C. Lee. Park attempted to stain Bonghan corpuscles using the trypan blue staining method and attempted to measure and analyze the electrical signals from Bonghan corpuscles of the intestines of rats [37]. He worked on three main research topics, measuring and analyzing the resting potential and spontaneous action potential using an intracellular recording method. He also observed the changing electrical potential by stimulation with various drugs, such as acetylcholine, modeled the electrical signals by BVP (Bonhoeffer-Van der Pol) modeling, and analyzed the findings by fractal theory. J. H. Choi, C. J. Choi, and Cho developed the electrophysiology of the primo vascular system after this research [43–45].

### 4.1. Measurement of the Resting Potential and Spontaneous Bioelectrical Potential of Primo Nodes and Vessels

Park recognized that the measurement of electrical signals was very important, because if Bonghan corpuscles and ducts are connected to each other and some liquid flows through the ducts, a driving force is necessary to deliver the liquid. According to this result, a resting potential and spontaneous action potential existed. They reported experimental values of −39.9 ± 15.5 mV and 1.2 ± 0.6 mV [37].

This result was highly significant as it was the first evidence that Bonghan corpuscles were composed of excitable cells and not types of fibrins and collagen fibers. In those days, almost every anatomical and histological expert insisted that Bonghan corpuscles and ducts are just collagen fibers and types of fibrins. So, the corpuscles and ducts are not only new structures but are nonfunctioning despite the fact that they may be new structures. However, his result showed that the spontaneous action potential was observed; thus, the corpuscle must have some special function.

**Figure 14:**
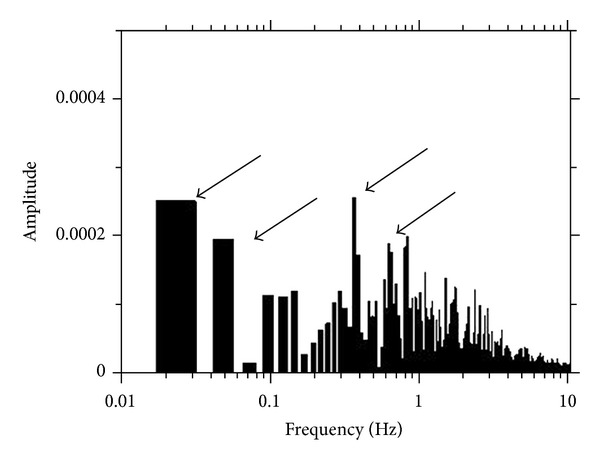
Fast Fourier transform (FFT) of the membrane potential. In this particular experiment, there were four values, 0.62–0.84 Hz, 0.36 Hz, 0.05 Hz, and 0.02 Hz, appearing above the broad background.

Moreover, when the electrical signals were analyzed, the primary wave groups were 0.62–0.84 Hz, 0.36 Hz, 0.05 Hz, and 0.02 Hz (Figure 14). It is interesting to note that this is similar to the *ㄱ* (geu), *ㄴ* (neu), and *ㄷ* (deu) wave forms reported by Kim in the 1960s [6, 24–27, 34].

Choi developed a more precise experimental setup and experiment that measured the electrical potential from Bonghan ducts [44]. He was an oriental medical doctor and a physicist who thought that Bonghan corpuscles and ducts were not related to the Kyungrak system. In addition, he hypothesized that Bonghan ducts were types of fibrin and were similar to lymphatic vessels by conducting a comparison experiment.

**Figure 15:**
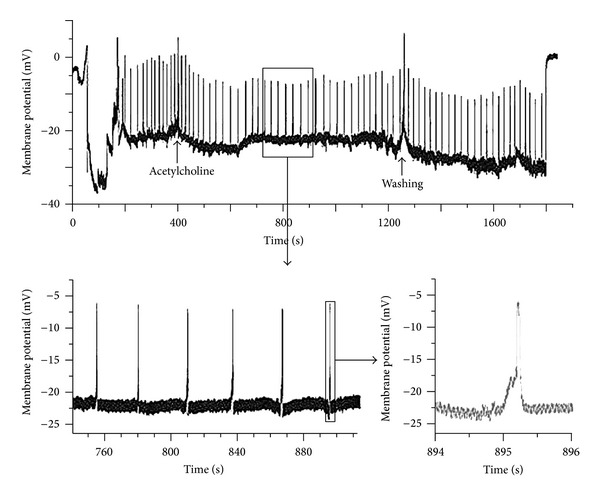
Spontaneous action potentials of the novel thread part. The resting potential was −22 mV; this is a quite unique value compared to that of other excitable cells. The bursting frequency decreased after applying acetylcholine and increased slightly after washing.

However, he observed the resting potential (*n* = 17) and spontaneous action potential (*n* = 2) of Bonghan ducts, reporting a resting potential of −10.0 ± 4.7 mV from cells embedded in the surfaces of Bonghan ducts. He concluded the bioelectrical signals from Bonghan ducts and lymphatic vessels were different and that it would be pointless to discuss a comparison with fibrins. This was an important result which overturned his previous hypothesis, representing the first report of the resting potential and spontaneous action potential of Bonghan ducts (Figure 15) [44, 46]. There was some discussion about revising the nomenclature to expand the study of the Bonghan system around that time (Table 2).

**Figure 16:**
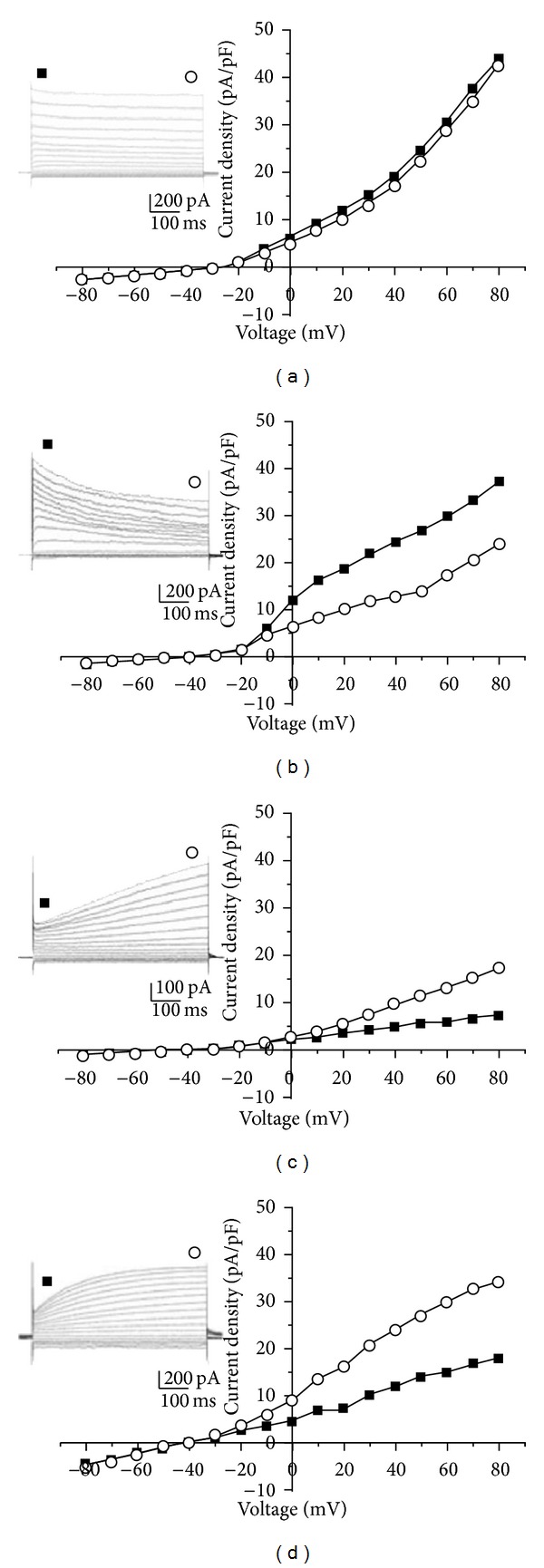
Four types of current-voltage (I.V) relationships recorded from cells in PN slices. I.V curves were obtained by depolarizing step pulses from −80 to 80 mV. (a) Type II.V relationships showing outward rectification. Note that the I.V relationships measured at 50 ms (solid squares) and 550 ms of the 600 ms current pulse (open circle) are identical. (b) Type III.V relationships showing outward rectification with the time-dependent activation of the outward current. (c) Type III I.V relationships showing outward rectification with a time-dependent and linearly activated outward current. Note the lower current density in this cell. (d) Type IV I.V relationships showing outward rectification with a time-dependent and hyperbolic increase in the outward current. The insets show the current traces for the I.V relationships in (a) and (d). The holding potential is −30 (a) and −40 mV (b and d). The largest outward tail current is seen in the current traces of the type IV cell. Scale bars for the insets are 10 *μ*m.

The intracellular recording method and the patch clamp method were used to measure the bioelectrical signals from Bonghan corpuscles and ducts by Choi [44]. He measured the electrical signals using a whole-cell slice-patch recording method for primo nodes and an intracellular recording method for primo vessels. His result reported a resting potential of –36.60 ± 1.38 mV of the primo nodes but no spontaneous action potential. Small round cells are most abundant in primo nodes. On the basis of the current-voltage (I-V) relationships and kinetics of the outward currents, the cells of primo nodes could be grouped into four types. Among these, type I cells were the majority (69%) [43]. He reported that the resting potential of primo vessels was 21.0 ± 2.2 mV and that there were two groups based on the resting potential (Type A (70%): −13.13 ± 0.66 mV and Type B (30%): −38.64 ± 2.96 mV). However, he reported that there were no properties of the spontaneous action potential of primo nodes and vessels, even if there were 2–4 cell groups (Figure 16) [43].

The Korea Institute of Oriental Medicine began research on the primo vascular system in 2009. Lee et al. reported a method of distinguishing between torn mesentery and primo vessels [47]. Cho reported that there was the spontaneous action potential of primo vessels on internal organs using an extracellular recording method. The value of the potential was different from the action potential of a pacemaker of intestines. There were two types of cell groups reported as well [45]. 

Summarizing the research results pertaining to the resting potential and spontaneous action potential of primo nodes and vessels, resting potential and spontaneous action potential of cells from primo nodes and vessels and several cell type groups were discovered.

This conclusion was similar to the report of B. H. Kim, who found that there were distinct bioelectrical signals from the tissues of Bonghan corpuscles and ducts, also finding that modern electrophysiological technology could increase the confidence in results pertaining to the properties of bioelectrical signals from the primo vascular system.

### 4.2. Electrophysiological Study of the Primo Vascular System by Responding Drugs

Park was interested in the changing bioelectrical signals of Bonghan corpuscles by drug stimulation after measuring the resting potential and spontaneous action potential [37].

This was direct evidence that the Bonghan system may be a circulatory system such as the cardiac vascular system and the lymphatic system, because if there were excitable cells of the Bonghan system that responded to drugs, the system could contribute to the functions of the living body, like nerves and muscles. He attempted to find the response of bioelectrical signals from Bonghan corpuscles using acetylcholine, pilocarpine, atropine, and nifedipine. Acetylcholine is a major neurotransmitter in an autonomic nervous system. It stimulates both nicotinic and muscarinic receptors and is a common drug used to stimulate muscles. Pilocarpine is a nonselective muscarinic acetylcholine receptor agonist. Atropine is a cholinergic receptor antagonist and a competitive nonselective antagonist at central and peripheral muscarinic acetylcholine receptors. Consequently, as the electrical signals of Bonghan corpuscles respond to these drugs, Bonghan corpuscles have muscarinic receptors which are controlled by the autonomic nerve system. The Bonghan system may be an autonomic-nerve-controlled system.

Nifedipine is L-type Ca^2+^ channel blocker. The fundamental features of the spike generated in smooth muscles are related to the activation of the L-type Ca^2+^ channel. Ca^2+^ must be available for muscle contraction. Therefore, if there are Ca^2+^ ion channels in Bonghan corpuscles, it could be important evidence that Bonghan corpuscles have smooth muscle-like characteristics, such as contractibility and cell relaxation.

**Figure 17:**
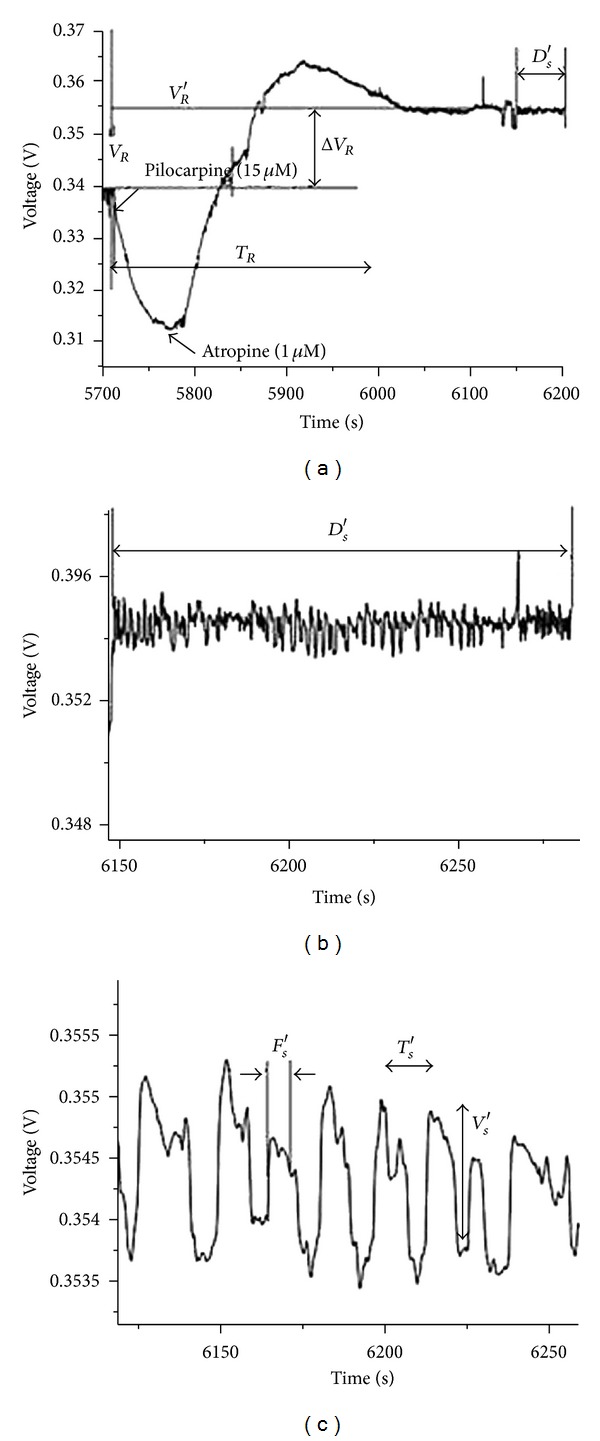
Effects of atropine on the resting potential and the spontaneous burst. (a) The resting potential dropped from *V*_*R*_ to *V*_*R*_′ and the decrease was Δ*V*_*R*_. The resting potential slowly recovered its original value by Δ*V*_*R*_. (b) The duration of the spontaneous burst (*D*_*s*_′). (c) The amplitude (*V*_*s*_′), period (*T*_*s*_′), and half width (*F*_*s*_′) of the spikes in the burst dramatically changed.

Park reported that the resting potential due to stimulation by acetylcholine was decreased to 50% and that the spike shape of the spontaneous action potentials completely changed [37]. Pilocarpine also changed the potential of Bonghan corpuscles similarly [48]. Although the corpuscles were stimulated by acetylcholine and pilocarpine, the resting potential of Bonghan corpuscles increased dramatically when stimulated by atropine (Figure 17) [37]. In the case of nifedipine stimulation, the resting potential also increased, though it decreased after stimulation with acetylcholine and pilocarpine. This was the first evidence of the existence of Ca^2+^ ion channels in the cell membranes of Bonghan corpuscles. 

This result confirms that the Bonghan system may have the functions of contractibility and relaxation of the circulatory system [37]. Particularly, Park asserted that the electrical signals of smooth muscle-like cells from Bonghan corpuscles have similar properties to those of the vascular smooth muscle reported by Bkaily [49].

J. H. Choi studied the responses to drugs. He used tetraethylammonium (TEA). TEA is known to block the K^+^ channel in nerves and Ca^2+^ channels are also blocked [50, 51].

Tetraethylammonium (TEA) dose-dependently blocked both the outward and inward current (IC_50_, 4.3 mM at ±60 mV). Under current clamp conditions, TEA dose-dependently depolarized the membrane potential (18.5 mV at 30 mM) with an increase in the input resistance. These results demonstrate for the first time that a TEA-sensitive current with limited selectivity to K^+^ contributes to the resting membrane potential in type I cells [52].

In summary, in the previous results of drug stimulation experiments, primo nodes are shown to have muscarinic receptors, allowing the primo vascular system to be controlled by the autonomic nerve system. Moreover, the system has the possibility of contractibility and relaxation function due to the Ca^2+^ ion channels. Moreover, the cells of the primo vascular system may be excitable given the K^+^ ion channels in the membranes of the cells. These results are in good agreement with the proteomics analyses of the primo vascular system [53].

## 5. Discussion

The results of the bioelectrical signals and electrophysiology research are summarized in Tables 3 and 4. 

The bioelectrical study of primo nodes and vessels simply focused on the measurement of the resting potential and spontaneous action potential initially, as nobody knew whether the cells from the primo vascular system had intrinsic potentials or not. Therefore, it is important to confirm the properties of the action potential of cells related to the basic physiology functions.

While examining the papers related to the bioelectrical study of the primo vascular system, there were slight differences in the results of the resting potential, action potential, and the drugs responses depending on the study. The research is not sufficient for a complete understanding of the functions of the primo vascular system in the body precisely, but the primo vascular system clearly has excitable cells that respond to some stimuli.

The physiological functions of the primo vascular system should be confirmed by investigating the properties of each cell in the system and by measuring the resting potential and the action potential of each cell type. If each cell type of the primo vascular system could be determined, we will be able to understand the signal delivery methods and the conduction system.

Ultimately, the purpose of an electrical signal analysis of the primo vascular system is to investigate whether the internal substances of the primo nodes and primo vessels can be circulated. In addition, it is necessary to conduct additional experiments to answer the questions pertaining to the existence of an exclusive signal conduction system of the primo system circulation, like the nervous system, and to find evidence supporting the circulating function of the primo system based on actual mechanical activity. Research areas requested in the future may include the following:

1. standardization of measured data by the standardization of techniques to measure the electrical signals of the primo vascular system;
2. characterizing the cells composing the primo vascular system;
3. investigating the electrical signal conduction and mechanical activity of the primo vascular system;
4. uncovering the signal conduction mechanism through a connection between the primo vascular system and internal organs materially;
5. investigating the control of the primo vascular system by external stimulation mechanisms such as medications and their interrelationships;
6. looking into the circulation of internal substances inside the primo vascular system.

In addition, it is recommended to adopt useful technology to measure two-dimension spatial signal conduction methods such as optical electrophysiological techniques, which have greatly progressed recently, in addition to previous techniques of measuring one-dimension signals from a single point [54].

Though research on the electrical signals of the primo system started in the 1960s, the reliability of the study results was limited due to the limitations of the measuring technology and short descriptions of the methods and data. However, several studies of the electrophysiological characteristics of the primo vascular system have been performed since the 2000s, and it will be interesting to watch the development in the field of the electrophysiology of the primo vascular system in the future.

In conclusion, there are many differenct type cells composed of primo vascular system; one is the excitable cell type and the other is the nonexcitable cell type.

In case of the excitable cell, the vascular smooth muscle like cell type [37], the intestinal smooth muscle like cell type [45] and the immune cell type are reported [46].

## Figures and Tables

**Table 1:** A comparison of electrophysiological characteristics between tissues.

	Neuron	Muscle		
		Skeletal	Cardiac	Smooth
Resting potential (mV)	−70	−80 to −90	−85 to −95	−50 to −60
Threshold potential (mV)	−55	−55	Spike potential	Spike potential
Action potential duration (ms)	1	2 to 5	200 to 400	10 to 50

**Table 2:** Revised nomenclature for the Bonghan system.

Before	After
Bonghan system	Primo vascular system
Bonghan duct	Primo vessel
Bonghan corpuscle	Primo node
Bonghan liquor	Primo fluid
Bonghan sanal	Primo microcell

**Table 3:** A comparison of the electrophysiological characteristics between primo nodes, primo vessels, neurons, and muscle cells.

	RP (mV)	APD (mV)	Drug-responsive	Reference
Neuron	−70	1	Y	[19]
Muscle				
Skeletal	−80 to −90	2 to 5	Y	[20]
Cardiac	−85 to −95	200 to 400	Y	[21]
Smooth	−50 to −60	10 to 50	Y	[22]
Primo node				
Skin	•	0.1	Y	[25]
Surface of a small intestine	−39.9 to 15.5	1.2 to 0.6	Y	[37]
Surface of a small intestine	−36.6 to 1.38	•	Y	[43]
Primo vessel				
Skin	•	0.1	Y	[25]
Surface of a small intestine	−10 to 4.7	20 40	•	[44]
Surface of a small intestine	A: −13.1 to 0.7B: −38.6 to 3.0	•	Y	[43]
Surface of a small intestine	•	A: 10.0 to 8.4B: 13.7 to 8.7	•	[45]

RP: resting potential; TP: threshold potential; APD: action potential duration (bioelectrical activity).

**Table 4:** The summarization of the electrophysiological properties of the primo vascular system.

Target	Methodology	Cell Type	Result	Reference
Primo node	Intracellular recording	Vascular smooth muscle like	Excitable (Muscarinic receptor, Ca^2+^ ion channel)	[37]
	Patch clamp, Current-voltage relationship	Four types	Nonexcitable cell (K^+^ ion channel for Type I)	[43]
	Extracelluar recording	Two types	Excitable cell (not smooth muscle like)	[45]

Primo duct	Intracellular recording	Smooth muscle Secreting cell Immune cell	Excitable Non-excitable	[44]
